# Effectiveness and Implementation of a Text Messaging mHealth Intervention to Prevent Childhood Obesity in Mexico in the COVID-19 Context: Mixed Methods Study

**DOI:** 10.2196/55509

**Published:** 2024-04-09

**Authors:** Ana Lilia Lozada-Tequeanes, Florence L Théodore, Edith Kim-Herrera, Armando García-Guerra, Amado D Quezada-Sánchez, Rocio Alvarado-Casas, Anabelle Bonvecchio

**Affiliations:** 1 National Institute of Public Health Instituto Nacional de Salud Pública de México Cuernavaca Mexico

**Keywords:** effectiveness, feasibility, mHealth, SMS text message, mixed methods, infant obesity, physical activity, healthy feeding, children, COVID-19, Mexico

## Abstract

**Background:**

Promoting physical activity (PA) and healthy feeding (HF) is crucial to address the alarming increase in obesity rates in developing countries. Leveraging mobile phones for behavior change communication to encourage infant PA and promote HF is particularly significant within the Mexican context.

**Objective:**

This study aims to explore the effectiveness and feasibility of mHealth interventions aimed at promoting PA and HF among primary caregivers (PCs) of Mexican children under the age of 5 years. Additionally, the study aims to disseminate insights gained from intervention implementation amidst the COVID-19 pandemic and assess the potential of behavior change mHealth interventions on a broader population scale.

**Methods:**

NUTRES, an mHealth intervention, underwent an effectiveness-implementation hybrid trial. Over 36 weeks, participants in the intervention group (IG), totaling 230 individuals, received approximately 108 SMS text messages tailored to their children’s age. These messages covered topics such as PA and HF and emphasized the significance of proper child nutrition amidst the COVID-19 pandemic. NUTRES participants were recruited from both urban and rural health units across 2 states in Mexico. Given the COVID-19 context, both baseline and follow-up surveys were conducted via mobile or fixed telephone. The evaluation of effectiveness and implementation used a mixed methods approach. Qualitative analysis delved into participants’ experiences with NUTRES and various implementation indicators, including acceptance, relevance, and coverage. Grounded theory was used for coding and analysis. Furthermore, difference-in-differences regression models were used to discern disparities between groups (comparison group [CG] versus IG) concerning knowledge and practices pertaining to infant PA and HF.

**Results:**

Of the total 494 PCs enrolled in NUTRES, 334 persisted until the end of the study, accounting for 67.6% (334/494) participation across both groups. A majority of PCs (43/141, 30.5%, always; and 97/141, 68.8%, sometimes) used the SMS text message information. Satisfaction and acceptability toward NUTRES were notably high, reaching 98% (96/98), with respondents expressing that NUTRES was “good,” “useful,” and “helpful” for enhancing child nutrition. Significant differences after the intervention were observed in PA knowledge, with social interaction favored (CG: 8/135, 5.9% vs IG: 20/137, 14.6%; *P*=.048), as well as in HF practice knowledge. Notably, sweetened beverage consumption, associated with the development of chronic diseases, showed divergence (CG: 92/157, 58.6% vs IG: 110/145, 75.9%; *P*=.003). In the difference-in-differences model, a notable increase of 0.03 in knowledge regarding the benefits of PA was observed (CG: mean 0.13, SD 0.10 vs IG: mean 0.16, SD 0.11; *P*=.02). PCs expressed feeling accompanied and supported, particularly amidst the disruption of routine health care services during the COVID-19 pandemic.

**Conclusions:**

While NUTRES exhibited a restricted impact on targeted knowledge and behaviors, the SMS text messages functioned effectively as both a reminder and a source of new knowledge for PCs of Mexican children under 5 years of age. The key lessons learned were as follows: mHealth intervention strategies can effectively maintain communication with individuals during emergencies, such as the COVID-19 pandemic; methodological and implementation barriers can constrain the effectiveness of mHealth interventions; and using mixed methods approaches ensures the complementary nature of results. The findings contribute valuable evidence regarding the opportunities and constraints associated with using mobile phones to enhance knowledge and practices concerning PA and HF among PCs of children under 5 years old.

**Trial Registration:**

ClinicalTrials.gov NCT04250896; https://clinicaltrials.gov/ct2/show/NCT04250896

## Introduction

Because of the short and long-term adverse effects of overweight/obesity on health and human capital, the World Health Organization (WHO) emphasizes its prevention early in life as a critical priority [1]. There is mounting evidence indicating that children from low socioeconomic backgrounds exhibit higher rates of overweight/obesity [2,3]. Mexico is particularly alarmed by the escalating prevalence of overweight/obesity among children under 5 years old, with the most recent national estimates projecting a combined rate of approximately 8% in 2022 [4]. Nevertheless, socioeconomically disadvantaged families are often more challenging for the health sector to engage with, and they may be less inclined to participate in programs promoting healthy behaviors [5]. Mobile health (mHealth) technologies and telecommunications present themselves as appealing low-cost interventions capable of reaching vast and remote populations [6].

In low- and middle-income countries [7], resources have been directed toward the development of mHealth interventions, including behavior change communication (BCC) strategies [8]. A 2019 systematic review [9] revealed that the most commonly used mHealth technology was SMS text messaging (60%). Additionally, there is evidence suggesting that eHealth and mHealth interventions are effective in promoting physical activity (PA) and healthy feeding (HF) in developing countries [10].

Hence, leveraging mobile phones for BCC interventions presents a promising opportunity to advocate for HF and PA practices among primary caregivers (PCs) of the Mexican child population. This is especially significant considering the rising and widespread use and accessibility of this technology, which has increased from 71.5% of the total population in 2015 to 79.2% in 2022 [11]. In particular, in emergency contexts where health services are disrupted and face-to-face information dissemination to the population is hindered, as was evident during the COVID-19 pandemic, leveraging innovative mHealth interventions becomes crucial. In this regard, testing and evaluating such interventions in emergency settings can significantly contribute to the existing evidence on mHealth. Moreover, it can serve to stimulate further research endeavors to complement and expand upon the findings of this study.

Hence, this research aimed to assess the effectiveness and implementation of an mHealth intervention, referred to as NUTRES, in promoting PA and HF practices among Mexican PCs of children under 5 years. A secondary objective was to document the lessons learned from implementing and evaluating an mHealth intervention during the COVID-19 pandemic.

## Methods

### Study Design

This study constitutes an effectiveness-implementation hybrid trial [12], incorporating elements from both effectiveness and implementation research. The trial is registered under the Trial Registration ID NCT04250896. We used a mixed methods approach [13], utilizing a “convergent advanced design” [14]. This design involved the simultaneous use of qualitative and quantitative methods, allowing for the triangulation of results from both methodologies. By combining statistical findings with insights gleaned from individuals’ real-life experiences, we aimed to gain a comprehensive understanding of the effects and implementation process of NUTRES.

### Eligibility Criteria and Recruitment of Participants

**Figure 1:**
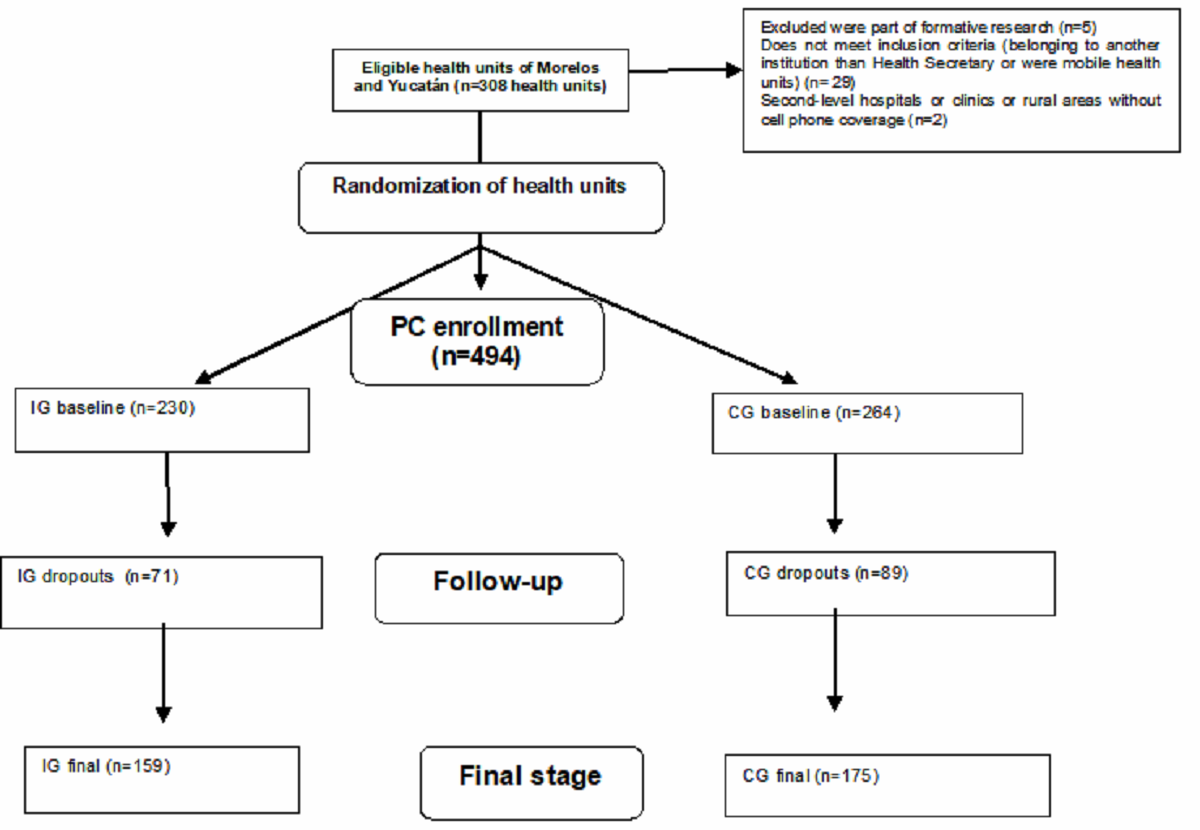
Flow diagram of NUTRES participants. CG: comparison group; IG: intervention group; PC: primary caregiver.

NUTRES participants were recruited from urban and rural health units situated in 2 states of Mexico: Morelos in the central region and Yucatán in the southern region. Randomization was conducted at the health unit level. Inclusion criteria comprised being a primary health care unit, being located in an area with access to a mobile phone network, having more than 50 registered users under 5 years, and having over 80% of the population with Spanish as their first language. A total of 308 eligible urban and rural health units from the 2 states were included in the study, assigned either to the intervention group (IG) or the comparison group (CG) (Figure 1).

The eligibility criteria for participants in both the IG and the CG were being the PC of an infant under 5 years, having ownership/access to any type of mobile phone, being able to speak and read Spanish, being aged 18 years or older, and being a resident in the coverage area of the participating health unit. Participants were recruited through 3 methods: (1) invitation from primary health providers, (2) snowball sampling, and (3) face-to-face encounters by field workers at health units. The CG had the same eligibility criteria for recruitment, except for the requirement of mobile phone ownership/access.

### Sample Size

#### Quantitative

Sample size calculations indicated that 100 participants per study group would yield 80% power at the .05 significance level to detect a 20%-point difference in the proportion of change for at least one outcome variable (eg, knowledge, attitudes, or practices related to infant PA or HF) between study groups, using a 2-sided test. These calculations accounted for a design effect ranging between 1.5 and 1.9 [15] and factored in a 20% dropout rate [16]. Notably, this sample size estimation did not consider states or areas as strata, thus precluding intergroup comparisons based on geographical regions.

#### Qualitative

The sample selection was purposive, with the aim of capturing a diverse range of experiences associated with NUTRES. Informants were chosen from both rural and urban areas, encompassing caregivers with children aged 0-23 months and 2-5 years, representing varying levels of interactivity. The use of 2-way SMS text messaging served as an indicator of their engagement with NUTRES (details on interactivity provided below).

### NUTRES Intervention

NUTRES is an mHealth BCC strategy designed to prevent childhood overweight/obesity. It achieves this by disseminating SMS text messages to PCs of children under 5 years, as well as to health personnel operating within primary unit services in 2 Mexican states. In this paper, we will primarily concentrate on the outcomes of the SMS text messaging interventions aimed at promoting infant PA [17] and HF [18]. For a comprehensive understanding of the development of NUTRES, readers are referred to previous publications [19,20].

In essence, the NUTRES intervention comprised 1- or 2-way SMS text messages, each containing fewer than 150 characters, disseminated to PCs over 9 months (equivalent to 36 weeks). Participants in the IG received approximately 108 SMS text messages, adjusted based on the child’s age. These SMS text messages addressed various aspects, including attitudes and practices concerning infant PA and HF. Additionally, SMS text messages addressing nutrition within the context of the COVID-19 pandemic were incorporated due to the emergency situation. The design of the SMS text message content was informed by extensive formative research [21], which aimed to identify both barriers and facilitators to the adoption of healthy practices within the target population. Every factor that could either facilitate or hinder behavior change was systematically addressed in line with the Theory of Planned Behavior [22,23]. This was accomplished through the dissemination of truthful information, practical tips, healthy recipes, challenges, and socioemotional support messages to PCs. All SMS text messages were meticulously aligned with the latest national [24] and international guidelines concerning infant PA and HF [17]. See Multimedia Appendix 1 for examples of SMS text messages delivered.

PCs who consented to participate in NUTRES were asked to provide details about the child’s name, age, and gender during registration. This information was used to personalize and tailor SMS text messages for each participant accordingly. NUTRES incorporated 2 types of SMS text messages: 1- and 2-way (the latter being sent following a response from PCs to the initial SMS text messages). On average, participants received approximately 3 SMS text messages per week. In addition to informational messages, a weekly positive socioemotional support SMS text message was dispatched to PCs, as research has demonstrated its effectiveness in fostering participation and maintaining interest [25]. These SMS text messages were programmed to be automatically delivered to PCs via the Rapid Pro platform [26], and interactivity, including responses from PCs, was recorded and tracked.

### Data Collection and Analysis

#### Overview

Data collection took place from September 2020 to September 2021. The quantitative component encompassed both the IG and the CG, involving both baseline and final assessments. Meanwhile, the qualitative component solely focused on the IG, conducted after 36 weeks of exposure to NUTRES. Both the qualitative and quantitative teams, consisting of approximately 4 members each, possessed extensive fieldwork experience and proficiency in communication technology. They underwent a week-long virtual training session via Zoom (Zoom Video Communications, Inc.).

#### Quantitative Data

Because of the COVID-19 context, both baseline and follow-up surveys were conducted via mobile or fixed telephone. The surveys covered a range of topics, including sociodemographic information, health status, and knowledge pertaining to infant PA and HF. Knowledge of recommended practices was assessed by querying respondents about the advantages, disadvantages, or known recommendations regarding PA and HF. Each answer was assigned a score based on its correctness, as outlined in Multimedia Appendix 2.

Furthermore, exposure to NUTRES was gauged by querying PCs regarding their receipt of the SMS text messages, as well as their perception of the usefulness and practicality of the messages. For instance, PCs were asked if they recall a specific message from NUTRES that they had implemented in practice, and their responses were recorded without any prompts or suggestions from the interviewer.

Data collection was conducted using the REDCap (Research Electronic Data Capture; Vanderbilt University) app, with daily verification and backup procedures in place. Following data collection, thorough exploration, cleaning, and recategorization processes were undertaken to prepare for descriptive statistics and basic comparative analyses between study groups. Topics of interest were examined and translated into knowledge and practice indices, with summary variables generated for each question. Practices were categorized into 3 equally sized tertiles for evaluation: the first tertile was termed “limited,” the intermediate tertile was termed “moderate,” and the tertile with higher values was termed “adequate.” Lastly, we used a double-difference approach [27] to evaluate the variance in the change of outcomes between the presence (IG) and absence (CG) of the intervention. A significance level of *P*<.05 was deemed significant. All statistical analyses adhered to an “intention-to-treat” principle [28] and were conducted using the Stata version 14.2 statistical package (StataCorp).

#### Qualitative Data

Interviews were conducted after the quantitative survey and delved into various topics, including opinions regarding NUTRES; technical issues encountered with receiving, reading, and responding to SMS text messages; overall impressions of the SMS text message content; behaviors encouraged by the messages; and the perceived impact of the intervention on the intention or ability to carry out recommended practices. Additionally, we explored 3 implementation indicators with PCs from the IG:

- “Acceptance” of NUTRES and behaviors promoted by SMS text messages.
- “Pertinency,” which examines the relevance of information and behaviors promoted by the SMS text messages in NUTRES.
- “Coverage,” which examines the extent to which PCs received the SMS text messages.

The first 2 implementation indicators were proposed by Proctor and colleagues [29], while the last one was by Peters and colleagues [30]. According to these authors, these indicators are crucial determinants of the success, in terms of both implementation and expected outcomes, of an intervention.

The interview guide underwent a pilot phase involving role-play to ensure its smooth flow and enable interviewers to practice its implementation. Telephone interviews typically lasted between 40 and 80 minutes, and they were recorded and transcribed verbatim. Following the principles of grounded theory [31], data analysis was conducted using a coding tree [31] to streamline the coding process. This analysis was performed utilizing NVivo 2020 (QSR International). See Multimedia Appendix 3 for the category tree used for coding.

### Ethical Approval and Consent

This study received approval from the Ethics, Research, and Biosafety Committees of the National Institute of Public Health (INSP) of Mexico, with the reference number CI 1547. All participants provided verbal informed consent to take part in the study.

## Results

### General Data About Design, Participants, and Sample Size

#### Quantitative

The total number of participants at baseline was 494 PCs, with 230 in the IG and 264 in the CG. Out of these, a total of 334 participants completed the study, comprising 118 from Morelos and 216 from Yucatán (Figure 1) [32]. The primary reasons for the loss to follow-up were the inability to locate the PC for final measurement, accounting for 9.1% (20/220) from the IG and 18.1% (41/227) from the CG, and the unwillingness of some participants to continue with the research, constituting 4.3% (8/187) from the IG.

The baseline characteristics of PCs revealed that the majority were young, with an average age of approximately 28 years. Most PCs were married or cohabiting and had a low level of education, with less than one-quarter having completed primary education. Additionally, at baseline, it was observed that the children of PCs from the CG were on average 3.7 months older than those from the IG (*P*<.001). Furthermore, PCs from the CG were less likely to report having a paid job compared with PCs from the IG (*P*<.004; Table 1). Baseline characteristics were found to be similar between PCs who continued in the study and those who were lost to follow-up. This is detailed in Multimedia Appendix 4, which presents the descriptive characteristics of individuals lost to follow-up versus those who completed the study.

**Table 1:** Sociodemographic characteristics of the primary caregivers (N=494) at baseline and final line, by study group (NUTRES, 2020-21).

Sociodemographic characteristics			Baseline				
			Comparison group (n=264)		Intervention group (n=230)		*P* value
**States of Mexico, n (%)**							.80
	Morelos	90 (34.1)		88 (38.3)			
	Yucatán	174 (65.9)		142 (61.7)			
**Area, n (%)**							.86
	Urban	162 (61.4)		148 (64.3)			
	Rural	102 (38.6)		82 (35.7)			
Age of primary caregiver, mean (SD)			27.5 (7.25)		27.1 (6.80)		.49
**Relationship to the child, n (%)**							.49
	Mother	259 (98.1)		226 (98.3)			
	Grandmother	1 (0.4)		2 (0.9)			
	Aunt/sister	3 (1.1)		1 (0.4)			
	Father/grandfather	0 (0)		1 (0.4)			
**Sex of child, n (%)**							.44
	Girl	126 (47.7)		104 (45.2)			
	Boy	138 (52.3)		125 (54.3)			
**Age of child (months), mean (SD)**			20.5(16.8)		16.8 (15.7)		.001
	<24, n (%)	165 (62.5)		164 (71.3)		.12	
	24-59, n (%)	99 (37.5)		66 (28.7)			
Marital status (married/free union), n (%)			232 (87.9)		191 (83.0)		.33
Schooling (basic or less), n (%)			10 (3.8)		27 (11.7)		.35
Employment with payment (last week) (yes), n (%)			22 (8.3)		40 (17.4)		.004
**Socioeconomic status^a^, n (%)**							.19
	Tertile 1	105 (39.8)		72 (31.3)			
	Tertile 2	90 (34.1)		67 (29.1)			
	Tertile 3	69 (26.1)		91 (39.6)			
Beneficiary/affiliation social program (yes), n (%)			64 (24.2)		61 (26.5)		.51

^a^Socioeconomic status (tercile 1 represents the lowest welfare conditions).

#### Qualitative

Twenty-four PCs from the IG were interviewed, with 12 participants from each state. These PCs had an average age of 30.2 years, with 14 participants having completed high school education or higher. The majority of interviewed PCs were homemakers, totaling 16 individuals. Multimedia Appendix 5 provides further details on the main characteristics of PCs interviewed for the NUTRES study conducted between 2020 and 2021. Interestingly, only a few differences were observed in the experiences reported by PCs of children aged 0-23 months and those aged 24-59 months, as well as between rural and urban areas. Consequently, qualitative findings and PC experiences are presented in a general manner.

### NUTRES Intervention Implementation

PCs expressed appreciation for the SMS text messages from NUTRES, noting that they valued their brevity and clarity. Informants unanimously agreed that the NUTRES strategy was “good,” “useful,” and “helpful.” PCs highlighted that NUTRES served as a “reminder” of previously acquired knowledge, while also introducing “new” information, particularly regarding the promotion of PA. PCs also remarked that the changing topics in the SMS text messages as their children grew made them feel supported and motivated to implement the recommendations, highlighting the acceptability and pertinence of the intervention (Textbox 1; Multimedia Appendix 6). Additionally, an overwhelming majority of PCs (96/98, 98%), expressed their desire to continue receiving NUTRES SMS text messages. However, some PCs raised concerns about sharing sensitive information, such as personal data, during the registration phase. Furthermore, a portion of PCs, specifically 32/145 (22%), reported encountering barriers to receiving SMS text messages from NUTRES. These barriers were damaged equipment (11/32, 34%) and issues related to connectivity or lack of mobile phone credit (7/32, 22%), indicating challenges in coverage (Table 2). Additionally, PCs indicated that they consistently use (43/141, 30.5%) and interact (27/140, 19.3%) with the 2-way SMS text message feature. Testimonies revealed that low interaction by PCs did not necessarily correspond to a lack of interest or rejection of NUTRES but rather to external circumstances such as a lack of credit or signal to receive SMS text messages, or a lack of awareness about the reply option (Textbox 1).

**Implementation indicators (English quotes) of NUTRES by primary caregivers exposed to NUTRES.**

**Acceptance**

- Well, I don't know, I say how....one year, I don't know...because I still don't feel as prepared for...as for...well...yes, well yes, I do feel prepared for my baby, but I still don't know how many things, so it does help me [#14]
- They make me feel calm [the messages]. (S) how do I explain it? They make me feel calm because they are helping me” (...) and it helped me a lot with the advice is reaching me (...) [#15].
- Mmm, well, it's a program that helps us, helps us complement what we already do at home for the children [#24].

**Pertinency**

- Since I am a first-timer, I felt that it helped me in the sense that, like right now, because of the Covid, there is no need for you to take, for example, to your health clinic, so there they told you how the processes that touched them...in what month could you give certain foods to your baby [#5].
- Yes, because they were at the age, they were perfectly fine at my son's age. Hey? forever! [#13].

**Coverage**

- Good. Well, in fact, we had to answer “Yes, No”, or if the goal was achieved or not, I tell him. There were times that the same and maybe I did not realize some messages because there was no coverage, they did not enter. Sometimes several days passed, and until I went out to some place that did exist, I was aware of the messages [#23]
- And I told him “it's that they don't reach me”, that only when I go to a higher part or I left here, they began to reach me. And I tell him, when I'm away, well, yes, I answer them, yes or...like no or something, well, the things they asked me [#21]
- Here in the town the current fails a lot, and the signal goes away...it's been like [P] four months now that the current goes out constantly here in the town and it's in parts [#6].
- Well, almost all of them because it was at the beginning when I began to receive the messages that they were going to charge me. And then, well, this...well, my recharge ran out very quickly, so to speak, and I preferred to just read them and put them into practice...Sometimes I would send them and say no, that I had to charge I don't know what...things like of money or something like that, and others if they let them answer [#14].
- What happens, that as there was a problem, that the telephones were lost. There came a time when this number that I have, my husband had it, and since we removed the chip, it disappeared [#5].

**Table 2:** Reception and reading of SMS text messages and the most useful format to put them into practice in primary caregivers participating in NUTRES.

Responses		Values (n=145)^a^
Difficulties receiving NUTRES SMS text messages (n=145), n (%)		32 (22.1)
**Reading of NUTRES SMS text messages (n=144), n (%)**		
	Always	96 (66.7)
	Sometimes	45 (31.3)
**Use of NUTRES SMS text messages (n=141), n (%)**		
	Always	43 (30.5)
	Sometimes	97 (68.8)
**Interactivity of NUTRES SMS text messages (n=140), n (%)**		
	Always	27 (19.3)
	Sometimes	71 (50.7)
**PCs^b^ reported barriers to receiving NUTRES SMS text messages (n=32), n/N (%)**		32/145 (22.1)
	Dropped telephone line, n (%)	3 (9.4)
	Lack of telephone signal, n (%)	7 (21.9)
	Change of phone number, n (%)	1 (3.1)
	Lack own mobile phone equipment, n (%)	3 (9.4)
	Damaged equipment, n (%)	11 (34.4)
	Lack of connectivity or mobile phone credit, n (%)	7 (21.9)
	Problem or difficulty was solved (yes), n (%)	11 (34.4)
**Reasons for not reading NUTRES SMS text messages (n=3), n/N (%)**		3/145 (2.1)
	Other (damaged or missing equipment), n (%)	3 (100.0)
**Reasons PCs did not implement NUTRES SMS text messages (n=1), n/N (%)**		1/145(0.7)
	Lack of time, n (%)	1 (100.0)
**Reasons PCs did not respond to NUTRES SMS text messages (n=42), n/N (%)**		42/145 (29.0)
	Lack of time and participants did not respond to SMS text messages within 12 hours, n (%)	7 (16.7)
	It is complicated, many issues, n (%)	1 (2.4)
	Information is missing, n (%)	2 (4.8)
	Other (eg, lack of own equipment, the economic burden for payment of phone line, error when sending responses, read SMS text messages later), n (%)	31 (73.8)
	Does not know/no comments, n (%)	1 (2.4)
**Perception of NUTRES (satisfied and very satisfied) (n=141), n/N (%)**		141/145 (97.2)
	Because is practical, n (%)	92 (65.2)
	A good resource in times of COVID-19, n (%)	10 (7.1)
	Received interesting messages, n (%)	83 (58.9)
	Other (eg, because of the interest they show toward the family, important messages for the health of babies), n (%)	7 (5.0)
**Reasons for dissatisfaction or some dissatisfaction with NUTRES (n=3), n/N (%)**		3/145 (2.1)
	Unclear or confusing information, n/N (%)	1/145 (0.7)
**Relevance of NUTRES (relevant/appropriate and highly relevant/very appropriate) (n=140), n/N (%)**		140/145 (96.6)
	It helps them with feeding and PA^c^ of their daughters and sons or with counseling of health providers, n (%)	87 (62.1)
	You can apply the recommendations in your daily life, n (%)	53 (37.9)
**Would you like to continue receiving SMS text messages from NUTRES? (Yes) (n=98), n (%)**		96 (98.0)
**Recommendations to improve NUTRES from PCs (n=159), n (%)**		
	Have a balance or credit/signal	28 (17.6)
	Have access to cell phone	3 (1.9)
	Know what to respond/clear instructions	6 (3.8)
	Have time	9 (5.7)
	Include images or videos (eg, WhatsApp)	16 (10.1)
	Be interesting	5 (3.1)
	Have training	8 (5.0)
	Other (eg, have a follow-up, include information about diseases, more examples, include foods from the region, make it face-to-face, more interaction, none)	51 (32.1)
	Do not know/no answer	63 (39.6)
**Most useful PA SMS text message format for PC (n=133), n (%)**		
	Challenge	61 (45.9)
	Socioemotional support	17 (12.8)
	Informative	21 (15.8)
	Recipes	2 (1.5)
	Tips	13 (9.8)
	With examples	15 (11.3)
	Links for more information	2 (1.5)
	Do not know/no answer	4 (3.0)
**Most useful HF^d^ SMS text message format for PC (n=142), n (%)**		
	Challenge	25 (17.6)
	Socioemotional support	20 (14.1)
	Informative	36 (25.4)
	Recipes	24 (16.9)
	Tips	21 (14.8)
	With examples	8 (5.6)
	Links for more information	2 (1.4)
	Do not know/no answer	5 (3.5)

^a^Totals may vary because of missing data.

^b^PC: primary caregiver.

^c^PA: physical activity.

^d^HF: healthy feeding.

The majority of PCs (92/141, 65.2%) reported that the SMS text messages were practical. Additionally, 57/145 (39.3%) PCs expressed a preference for the informative format over other formats, such as tips or challenges. PCs particularly appreciated the challenges related to PA, with 61/133 (45.9%) expressing appreciation because it involved other family members (Table 2). This aspect was particularly valued, especially in the context of COVID-19.

What I liked most about the SMS was that they sent advice, physical activity, not only for Mateo, but for the whole family and everything. And the same advice for the whole family to follow, not just for the baby, for my son

#3

PCs recalled messages regarding the importance of avoiding certain unhealthy food items for their children, such as sugar-sweetened beverages or sausages. These messages emphasized that these items were not suitable for children and were associated with the risk of early chronic diseases.

I remember that they recommended not to give him boxed juices, but to give him plain water or natural juice, that is, natural fruit juice

#2

The same, [NUTRES] would send us, for example (...) the sausages and all that, because it was not the right thing to do, because they have fat, if not, that it was better for them to eat chicken, pork, beef

#2

### NUTRES Intervention Effectiveness

At baseline, PCs from the IG exhibited greater knowledge and awareness regarding the risk of developing anxiety or depression due to lack of PA compared with the CG (4/179, 2.2% vs 13/149, 8.7%, respectively; *P*=.02). Following the intervention, significant differences between the 2 groups were observed, with the IG showing higher rates of favoring social interaction and integration in relation to knowledge of PA (CG: 8/135, 5.9% vs IG: 20/137, 14.6%; *P*=.048). Regarding HF practices, significant differences were observed between the IG and the CG in recognizing that diabetes and chronic diseases can develop as a consequence of sugar-sweetened beverage consumption (CG: 92/157, 58.6% vs IG: 110/145, 75.9%; *P*=.003; Table 3). However, in the difference-in-differences model, there was no significant improvement observed in the practice of infant PA and HF (*P*>.05; Table 4). The only significant increase between the study groups was observed in terms of knowledge about the benefits of PA (CG: mean 0.13, SD 0.10 vs IG: mean 0.16, SD 0.11; *P*>.02; Table 4). Nevertheless, several PCs reported improvements in their perceived control and intention related to some of the behaviors promoted by NUTRES, although detailed data on this aspect are not shown.

Well, as far as I know, I used to give my older children juice...soda, and [now] I don’t want to give her, I mean, any sweets

#16

**Table 3:** Knowledge and practices about infant physical activity and healthy feeding from primary caregivers: baseline versus final line (NUTRES, 2020-21).^a^

Knowledge and practice			Baseline			Final line		
			Comparison group (n=264)	Intervention group (n=230)	*P* value	Comparison group (n=175), %	Intervention group (n=159), %	*P* value
**Physical activity (PA^b^), n/N (%)**								
	**Main benefits of PA identified**		177/264 (67.0)	158/230 (68.7)	.43	135/171 (78.9)	137/157 (87.3)	.09
		Strengthens muscles and bones	103/177 (58.2)	91/158 (57.6)	.92	76/135 (56.3)	76/137 (55.5)	.89
		Strengthens the immune system	45/177 (25.4)	21/158 (13.3)	.17	28/135 (20.7)	19/137 (13.9)	.16
		Improves cognitive function, school performance, or both	42/177 (23.7)	43/158 (27.2)	.55	30/135 (22.2)	30/137 (21.9)	.94
		Promotes relaxation and well-being and improves sleep patterns	11/177 (6.2)	17/158 (10.8)	.18	15/135 (11.1)	19/137 (13.9)	.56
		Helps to prevent chronic diseases	44/177 (24.9)	26/158 (16.5)	.07	25/135 (18.5)	37/137 (27.0)	.09
		Helps to improve chronic disease control	35/177 (19.8)	21/158 (13.3)	.32	16/135 (11.9)	19/137 (13.9)	.62
		Helps to avoid anxiety/depression	3/177 (1.7)	4/158 (2.5)	.67	3/135 (2.2)	4/137 (2.9)	.70
		Promotes socialization and social interaction	12/177 (6.8)	14/158 (8.9)	.47	8/135 (5.9)	20/137 (14.6)	.048
		Promotes water consumption (improves hydration)	1/177 (0.6)	—^c^	.34	1/135 (0.7)	3/137 (2.2)	.33
		Others (less constipation, promotes growth, gives them energy, they express themselves better)	16/177 (9.0)	16/158 (10.1)	.83	6/135 (4.4)	4/137 (2.9)	.56
		Do not know/answer	1/177 (0.6)	—	.36	1/135 (0.7)	2/137 (1.5)	.53
**Main consequences of not doing PA**			179/264 (67.8)	149/230 (64.8)	.83	132/171 (77.2)	129/157 (82.2)	.38
		Decreased flexibility and weak muscles/bones	39/179 (21.8)	41/149 (27.5)	.32	31/132 (23.5)	40/129 (31.0)	.30
		Weak immune system	32/179 (17.9)	18/149 (12.1)	.40	25/132 (18.9)	19/129 (14.7)	.38
		Poor cognitive function/school performance	9/179 (5.0)	11/149 (7.4)	.48	20/132 (15.2)	12/129 (9.3)	.18
		Stress, annoyance, sadness, and insomnia	11/179 (6.1)	14/149 (9.4)	.31	10/132 (7.6)	12/129 (9.3)	.55
		Increased risk of chronic disease	131/179 (73.2)	92/149 (61.7)	.17	79/132 (59.8)	85/129 (65.9)	.37
		Increased risk of anxiety or depression	4/179 (2.2)	13/149 (8.7)	.02	4/132 (3.0)	9/129 (7.0)	.14
		Less socialization and social interaction	9/179 (5.0)	16/149 (10.7)	.11	13/132 (9.8)	18/129 (13.9)	.42
		Others (fatigue, poor oxygenation, become lazy, or sedentary)	8/179 (4.5)	10/149 (6.7)	.49	3/132 (2.3)	3/129 (2.3)	.97
		Do not know/answer	2/179 (1.1)	1/149 (0.7)	.67	1/132 (0.8)	2/129 (1.5)	.54
**PA recommendations in children <5 years**			56/264 (21.2)	49/229 (21.4)	.35	48/171 (28.1)	29/157 (18.5)	.14
		At least 3 hours per day (180 minutes/day)	2/56 (3.6)	2/49 (4.1)	.63	2/48 (4.2)	2/29 (6.9)	.58
**Screen time recommendations for children <5 years**			88/264 (33.3)	106/229 (46.3)	.30	90/171 (52.6)	85/157 (54.1)	.91
		<30 minutes/day	27/88 (30.7)	34/106 (32.1)	.59	33/90 (36.7)	33/85 (38.8)	.77
		<60 minutes/day	31/88 (35.2)	37/106 (34.9)		35/90 (38.9)	35/85 (41.2)	N/A^d^
		<2 hours/day	15/88 (17.0)	13/106 (12.2)		12/90 (13.3)	10/85 (11.8)	N/A
		Is avoided	12/88 (13.6)	12/106 (11.3)		7/90 (7.8)	4/85 (4.7)	N/A
		It depends on the age	2/88 (2.3)	2/106 (1.9)		1/90 (1.1)	0 (0)	N/A
		Other	—	4/106 (3.8)		2/90 (2.2)	2/85 (2.4)	N/A
		Do not know/answer	1/88 (1.1)	4/106 (3.8)		0 (0)	1/85 (1.2)	N/A
**Healthy feeding**								
	Identifies that it is important to include vegetables in the dishes’ preparation (vegetable consumption)		28/264 (10.6)	26/230 (11.3)	.86	159/175 (90.9)	146/159 (91.8)	.78
	Identifies the consumption of natural water as recommended		36/264 (13.6)	28/230 (12.2)	.80	11/175 (6.3)	19/159 (11.9)	.13
	Identifies the consumption of sugar-sweetened beverages as not recommended		6/264 (2.3)	5/230 (2.2)	.94	8/175 (4.6)	3/159 (1.9)	.13
	Identifies the consumption of ultra-processed products (eg, cupcakes, cookies) as not recommended		—	1/230 (0.4)	.30	3/175 (1.7)	4/159 (2.5)	.63
	**Consequences of an unhealthy feeding**							
		Undernourishment	196/264 (74.2)	162/224 (72.3)	.63	145/175 (82.9)	126/159 (79.2)	.41
		Overweight or obesity	71/264 (26.9)	64/224 (28.6)	.61	46/175 (26.3)	57/159 (35.8)	.13
		Respiratory diseases	21/264 (8.0)	16/224 (7.1)	.76	15/175 (8.6)	9/159 (5.7)	.28
		Musculoskeletal or skin problems	2/264 (0.8)	2/224 (0.9)	.88	16/175 (9.1)	3/159 (1.9)	.002
		Sleep disturbances, discouragement and tiredness, difficulty practicing a physical activity or any activity, learning difficulties	9/264 (3.4)	7/224 (3.1)	.83	7/175 (4.0)	4/159 (2.5)	.58
		Diabetes, hypertension, and hypercholesterolemia	25/264 (9.5)	25/224 (11.2)	.44	17/175 (9.7)	20/159 (12.6)	.48
		Candies and soft drinks are identified as prizes for life	0 (0)	1/224 (0.4)	.20	0 (0)	1/159 (0.6)	.32
		Harmful habits are established that are for life	0 (0)	1/224 (0.4)	.20	0 (0)	1/159 (0.6)	.32
		Others (eg, bulimia and anorexia, gastritis, diarrhea and vomiting, lack of appetite, dehydration, getting sick in general)	14/264 (5.3)	8/224 (3.6)	.23	1/175 (0.6)	2/159 (1.3)	.46
		Do not know/answer	14/264 (5.3)	13/224 (5.8)	.79	5/175 (2.9)	8/159 (5.0)	.31
	**Consequences of sugar-sweetened beverage consumption**					157/171 (91.8)	145/157 (92.4)	.67
		Undernourishment	14/215 (6.5)	13/185 (7.0)	.90	14/171 (8.2)	10/144 (6.9)	.46
		Overweight or obesity	96/215 (44.7)	97/187 (51.9)	.33	75/157 (47.8)	67/145 (46.2)	.79
		Respiratory diseases or diarrhea	5/215 (2.3)	3/185 (1.6)	.64	4/157 (2.5)	3/143 (2.1)	.81
		Musculoskeletal or skin problems	10/215 (4.7)	11/185 (5.9)	.62	17/157 (10.8)	8/145 (5.5)	.10
		Sleep disturbances, discouragement and tiredness, difficulty practicing a physical activity or any activity, learning difficulties	5/215 (2.3)	2/185 (1.1)	.92	13/157 (8.3)	2/143 (1.4)	.01
		Diabetes, hypertension, and hypercholesterolemia	139/215 (64.7)	116/187 (62.0)	.65	92/157 (58.6)	110/145 (75.9)	.003
		Candies and soft drinks are identified as prizes for life	0 (0)	1/185 (0.5)	.21	0 (0)	2/145 (1.4)	.18
		Harmful habits are established that are for life	2/215 (0.9)	2/185 (1.1)	.90	0 (0)	3/145 (2.1)	.12
		Others (eg, kidney problems, addiction, hyperactivity, stomach ache)	21/215 (9.8)	20/185 (10.8)	.84	4/157 (2.5)	5/145 (3.4)	.56
		Do not know/answer	1/215 (0.5)	2/185 (1.1)	.47	2/157 (1.3)	0 (0)	.31

^a^Totals may vary because of missing data.

^b^PA: physical activity.

^c^Not available.

^d^N/A: not applicable.

**Table 4:** Changes in knowledge and practices about infant physical activity and healthy feeding among primary caregivers in NUTRES.

Outcomes of interest			Baseline		Postintervention			*P* value
			Comparison group (n=264)	Intervention group (n=230)	Comparison group (n=175)	Intervention group (n=159)	Estimated change, double difference^a^	
**Knowledge**								
	**PA^b,c,d^**							
		PA concept, mean (SD)	0.26 (0.18)	0.3 (0.18)	0.28 (0.17)	0.27 (0.15)	–0.05	.04
		PA benefits, mean (SD)	0.13(0.11)	0.12 (0.11)	0.13 (0.10)	0.16 (0.11)	0.037	.02
		PA recommendations, mean (SD)	0.04 (0.10)	0.05 (0.10)	0.05 (0.09)	0.03 (0.06)	–0.024	.07
		Screen time recommendations (adequate), n (%)	88 (33.3)	106 (46.1)	90 (51.4)	85 (53.5)	N/A^e^	<.001
**Healthy feeding^b^, mean (SD)**								
		Healthy menu	0.18 (0.07)	0.19 (0.07)	0.19 (0.07)	0.21 (0.10)	0.014	.19
		Unhealthy feeding	0.25 (0.12)	0.25 (0.14)	0.26 (0.15)	0.29 (0.16)	0.025	.21
		Healthy drinks	0.25 (0.13)	0.26 (0.12)	0.25 (0.12)	0.26 (0.11)	0.001	.94
		Unhealthy drinks	0.14 (0.07)	0.14 (0.06)	0.14 (0.08)	0.15 (0.09)	0.013	.21
**Practices**								
	PA^b,c^, mean (SD)		5.30 (3.28)	4.94 (3.20)	8.65 (3.50)	7.67 (3.50)	–0.758	.08
	**PC^f^ of children between 6 and 23.9 months, mean (SD)**		4.81 (3.15)	4.60 (3.03)	9.68 (3.08)	8.28 (3.68)	–1.175	.10
		Adequate, n/N (%)	38/102 (37.3)	25/88 (28.4)	25/66 (37.9)	13/61 (21.3)	N/A	N/A
		Moderate, n/N (%)	19/102 (18.6)	13/88 (14.8)	24/66 (36.4)	21/61 (34.4)	N/A	N/A
		Limited, n/N (%)	45/102 (44.1)	50/88 (56.8)	17/66 (25.8)	27/61 (44.3)	N/A	N/A
	**PC of children ≥24 months, mean (SD)**		7.53 (3.05)	7.84 (3.14)	9.0 (2.22)	8.58 (2.58)	0.705	.30
		Adequate, n/N (%)	31/95 (32.6)	22/65 (33.8)	21/63 (33.3)	16/49 (32.7)	N/A	N/A
		Moderate, n/N (%)	33/95 (34.7)	20/65 (30.8)	22/63 (34.9)	14/49 (28.6)	N/A	N/A
		Limited, n/N (%)	31/95 (32.6)	23/65 (35.4)	20/63 (31.7)	19/49 (38.8)	N/A	N/A
	**Healthy feeding^g^, mean (SD)**		10.22 (3.09)	10.72 (2.69)	10.23 (2.32)	10.77 (2.25)	0.029	.96
		Adequate, n (%)	31/99 (31.3)	24/66 (36.4)	22/65 (33.8)	16/50 (32.0)	N/A	N/A
		Moderate, n (%)	34/99 (34.3)	21/66 (31.8)	18/65 (27.7)	20/50 (40.0)	N/A	N/A
		Limited, n (%)	34/99 (34.3)	21/66 (31.8)	25/65 (38.5)	14/50 (28.0)	N/A	N/A

^a^Difference-in-differences models adjusted for the baseline age of the child and maternal employment.

^b^Applied to all participants.

^c^An index of PA and healthy feeding practices was calculated, whose score was divided into tertiles to obtain the categories of “adequate,” “moderate,” and “limited” practices according to children’s age (annex 6).

^d^PA: physical activity.

^e^N/A: not applicable.

^f^PC: primary caregiver.

^g^Applied only to PC of children >24 months.

## Discussion

### Principal Findings

To our knowledge, this study represents the first report on the feasibility of implementation and effectiveness of an mHealth intervention aimed at preventing childhood overweight/obesity in Mexico within the context of COVID-19. This research offers valuable insights into the implementation process of NUTRES and its impact on expected outcomes. Although NUTRES demonstrated limited impact on target knowledge and behaviors, the SMS text messages served as reminders or sources of new knowledge among PCs of Mexican children under 5 years.

The absence of significant effects observed in this study may be partially attributed to methodological adaptations necessitated by the COVID-19 context, such as recruitment and data collection conducted via telephone. However, it is conceivable that NUTRES could have served to mitigate the impact of the pandemic on the participating population. Given that the COVID-19 pandemic exacerbated food insecurity and contributed to increased consumption of ultra-processed products, along with physical inactivity during lockdowns, the intervention may have provided some degree of protection to the population involved [33]. Furthermore, the COVID-19 pandemic severely disrupted health services, significantly limiting the population’s access to essential nutritional counseling [34].

Additionally, the lack of significant effects could be attributed to the high rate of loss to follow-up, which, although not markedly different between the study groups (71/230, 30.9%, loss in the IG and 89/264, 33.7%, loss in the CG), represented a loss to follow-up exceeding 20% (160/494, 32.4%) [35,36]. However, considering a sample size of 150 participants per study group and assuming a correlation of 0.6 between repeated measures within participants, our analysis sample achieved an approximate power of 83% to detect a difference of 0.15 in the change of proportions [37]. Nevertheless, it is plausible that the registration and follow-up process, which involved requesting contact information such as the child’s name or age, may have discouraged the participation of PCs due to concerns about privacy and security. To address this issue, other studies have implemented strategies such as providing payments or reminders, resulting in a high retention rate of over 80% [38]. Such approaches could potentially enhance participant engagement and retention in future interventions.

Another important consideration to mention is that, due to the nature of the intervention, access to and use of a mobile phone was a prerequisite for inclusion in the IG. This aspect could introduce potential selection bias [39], especially considering that mobile phone ownership was not a requirement for the CG and mobile phone ownership might be correlated with income level. However, this potential bias was addressed in the analysis by adjusting for paid maternal employment (40/230, 17.4%, in the IG vs 22/264, 8.3%, in the CG), among other baseline characteristics. Adjusting for these baseline characteristics helps mitigate the potential influence of confounding factors on the study outcomes.

Previous studies have not consistently shown significant effects of exposure to BCC mHealth interventions on the intended audience [8]. In this trial, although 96/144 (66.7%) PCs always read NUTRES SMS text messages, only 43/141 (30.5%) always used the information provided. However, based on PC testimonies, NUTRES served as a reliable source of recommendations or reminders for child care, which they incorporated into their practices. Simultaneously, these recommendations empowered them within their immediate family and social environment. Furthermore, while PC narratives expressed some enthusiasm, the limitations of conducting telephone interviews prevented the observation of informants’ physical responses, hindering further exploration and discussion of their enthusiasm. Therefore, future mHealth interventions should incorporate features to enhance engagement over time, using various methods such as incentives, push notifications, and other interactive elements. These strategies could help sustain interest and participation in the intervention among the target audience.

The results from both the qualitative and quantitative components of NUTRES are consistent with the lack of effectiveness observed in other studies regarding the impact of mHealth interventions on PC practices related to PA and HF. For example, a study conducted in China found that a weekly SMS text messaging intervention over 12 months had no significant effect on infant feeding practices [40]. It could therefore be speculated that the 9-month duration of exposure in NUTRES may have been insufficient to bring about changes in infant HF practices. However, NUTRES did demonstrate an improvement in PC knowledge regarding some benefits of PA. Indeed, PCs emphasized in interviews that family challenges played a crucial role in guiding and supporting them in implementing the recommendations provided by NUTRES. While the effectiveness of NUTRES could not be conclusively demonstrated, the mixed methods methodology used in this study identified that the obstacles to implementation were primarily external to NUTRES and largely technological in nature. Similarly, other studies conducted in Mexico have documented persistent barriers to mHealth implementation, such as the cost of mobile phones, lack of network infrastructure, and low digital literacy [41]. These barriers to implementing mHealth interventions may diminish the potential impact of smaller-scale studies and hinder their scale-up [41].

Our findings suggest that multicomponent and multichannel interventions have greater potential to promote behavior change compared with single-channel multitheme interventions. While the original design of NUTRES aimed for health providers to reinforce the intervention topics among PCs, this approach was not feasible due to the disruption of health services during the COVID-19 pandemic [42,43]. Nevertheless, an mHealth intervention could serve as a promising channel to maintain contact when face-to-face interaction is not feasible, complementing other remote interventions such as telemedicine [44,45].

One strength of this study is its effectiveness-implementation hybrid trial with an advanced convergent design, which enabled the comparison and interpretation of quantitative and qualitative results. Using a mixed methods approach, we were able to comprehensively observe the effects of the intervention and implementation process [13]. For example, our study revealed that the low interaction with SMS text messages by PCs did not necessarily indicate a lack of interest in NUTRES. Instead, it was often attributed to external factors such as poor signal reception, economic constraints, and other challenges. However, despite these insights, we were generally unable to demonstrate a significant effect of the NUTRES intervention on the target behaviors. Nevertheless, previous studies [46] investigating the usefulness of SMS text messages, as reported by PCs, suggest that valuable behavioral changes could potentially be achieved in the long term. Additionally, our difference-in-differences models were adjusted for child age and maternal employment, which were variables that differed between the groups at baseline. This adjustment allowed us to estimate the specific effect of the intervention. Furthermore, the double-difference approach accounts for time-invariant observed factors (eg, education) and unobserved variables (eg, motivation to comply) [27], providing a more robust analysis of the intervention’s impact.

### Lessons Learned

The positive feedback received through both quantitative and qualitative data aligns with findings from another mHealth intervention [47,48]. A systematic review conducted in 2019 [9] identified the top 3 barriers among participants, which included signal coverage, lack of equipment (unaffordable phones), and technology gap (limited knowledge of how to use a phone). In this study, despite the participants’ positive perceptions, similar challenges were encountered in terms of interaction with SMS text messages. For instance, some participants reported that SMS text messages were blocked, requiring them to contact their mobile operator to resolve the issue, which they were often unaware of. Additionally, other reasons for not receiving the SMS text messages were dropped lines, being out of coverage area, or changes in phone numbers (15/32, 47%). When participants did not respond to the SMS text messages within 12 hours, they were removed from the screen (7/42, 17%), and a significant portion of participants dropped out of the study (160/494, 32.4%). Overcoming such barriers is crucial to increase exposure to the intervention, thereby enhancing its impact and sustainability [49].

Based on the results presented here, this study provides valuable insights that can serve as a foundation for further research using mHealth interventions. The advantages of mHealth interventions, including broad coverage, cost-effectiveness, and feasibility of implementation in the population, underscore the potential for future studies in this area. It is suggested that future research should focus on addressing the identified challenges to improve the effectiveness, efficiency, and scalability of interventions. Some potential areas for improvement are (1) using WhatsApp (Facebook, Inc.) instead of SMS text messages (or in addition to SMS text messages), as it is a useful and accepted medium among the target population; (2) using informative material to recruit PCs; (3) improving SMS text message reception; (4) clarifying that SMS text message interaction has no cost; and (5) including resubscription for loss/change of mobile phone (Table 2).

Another valuable lesson learned is the importance of engaging key health care providers [50] and identifying informal strategies to recruit participants during emergency situations [33]. Furthermore, we have observed that mHealth interventions during lockdowns may represent the sole means of supporting socioeconomically vulnerable populations, who were often the hardest hit [51].

### Conclusions

The findings contribute to the existing evidence on the opportunities and limitations associated with mHealth interventions for BCC aimed at enhancing knowledge and practices related to PA and HF among caregivers of infants. Integrating mobile phone technology into BCC interventions facilitates the promotion of healthy lifestyles, particularly during emergency situations and among vulnerable populations. Moreover, the integration of effectiveness and implementation findings is valuable for optimizing mHealth interventions in particular settings, enhancing their sustainability, and fostering their application in diverse contexts [52].

## Appendix

Multimedia Appendix 1 Examples of SMS text messages delivered by mobile phone to primary caregivers’ of the intervention group of NUTRES, 2020-21.

Multimedia Appendix 2 Index of knowledge and practices on topics of interest, NUTRES.

Multimedia Appendix 3 Category tree for coding testimonies of primary caregivers of NUTRES, 2021.

Multimedia Appendix 4 Sociodemographic characteristics of primary caregivers at baseline and lost to follow-up. NUTRES, 2020-21.

Multimedia Appendix 5 Main characteristics of primary caregivers interviewed. NUTRES, 2020-21.

Multimedia Appendix 6 Implementation indicators of NUTRES by primary caregivers exposed to NUTRES.

## Abbreviations

BCC: behavior change communication
CG: comparison group
HF: healthy feeding
IG: intervention group
INSP: National Institute of Public Health
mHealth: mobile health
PA: physical activity
PC: primary caregiver
REDCap: Research Electronic Data Capture
WHO: World Health Organization
