# Supplementary material for: Effectiveness and Implementation of a Text Messaging mHealth Intervention to Prevent Childhood Obesity in Mexico in the COVID-19 Context: Mixed Methods Study
Source: JMIR Mhealth Uhealth. 2024 Apr 9;12:e55509. doi: 10.2196/55509 (PMC11005909; doi:10.2196/55509)
Supplement: Multimedia Appendix 1 [file mhealth_v12i1e55509_app1.docx]

Multimedia Appendix 1. **Examples of SMS´s delivered by mobile telephone to primary caregivers’ participants of intervention group of NUTRES, 2020-21.**

| Group of age of child under 5y old | SMSs of PA | SMSs of HF |
| --- | --- | --- |
| 0 a 6 months | Desde que tu bebé nace es importante pasar tiempo estimulando y moviendo su cuerpo ¡La actividad física o ejercicio le permitirá crecer y desarrollarse mejor!  ¡Hola! Cuando @cont.bebenom rueda en la cama o juega levantando sus piernitas, mejora la coordinación de su cuerpo ¡30 minutos al día son suficientes! | N/A |
| In English | From the moment your baby is born it is important to spend time stimulating and moving his body. Physical activity or exercise will allow him to grow and develop better!  Hello! When @cont.bebenom rolls around in bed or plays by raising his little legs, he improves his body coordination. 30 minutes a day is enough! | -- |
| 6 a 24 months | ¡Mamá! La actividad física o ejercicio es importante para el desarrollo físico y mental de tu bebé. Te enviaremos consejos para que sea mucho más divertido.  Pasar tiempo frente a la tele o celular daña el cerebro de @cont.bebenom. Motívale a gatear, a atrapar la pelota o agacharse ¡30 minutos al día son suficientes!  Recuerda cumplir el reto de jugar con @cont.bebenom a encontrar el juguete escondido | Cada día @cont.bebenom aprende de ti, prueba ofreciéndole 1 verdura en cada una de sus comidas, en nuevas presentaciones y sabores ¡Serán sus favoritas!  Recuerda cumplir con el reto de preparar comidas con verduras, para @cont.bebenom, incluyendo al menos 1 verdura al día |
| In English | Mother! Physical activity or exercise is important for your baby's physical and mental development. We will send you tips to make it much more fun.  Spending time in front of the TV or cell phone damages @cont.bebenom's brain. Motivate him to crawl, catch the ball or bend down. 30 minutes a day is enough!  Remember to complete the challenge of playing with @cont.bebenom to find the hidden toy | Every day @cont.bebenom learns from you, try offering him 1 vegetable in each of his meals, in new presentations and flavors. They will be his favorites!  Remember to meet the challenge of preparing meals with vegetables, for @cont.bebenom, including at least 1 vegetable a day |
| 24-59 months | Reto: esta semana pide al papá, hermano, primo, abuelito o tío de @cont.bebenom que jueguen activamente juntos y pasen momentos de diversión  ¡Hola! ¿Pudiste cumplir el reto de que el papá, hermano, primo, abuelito o tío de @cont.bebenom jugaran activamente juntos? Responde SÍ o NO a este mensaje  @cont.mamanom los niños y niñas de 2 a 5 años deben realizar actividad física o ejercicio hasta alcanzar 3 divertidas horas de juego a lo largo del día | Reto: esta semana preparale a @cont.bebenom comidas con verduras, por ejemplo calabaza, nopales, elotes o chayote. Intenta incluir al menos 1 verdura al día  ¡Hola @cont.mamanom! Una excelente opción para @cont.bebenom es darle verduras de temporada, porque son más frescas y económicas  Hola @cont.mamanom ¿Pudiste incluir verduras de temporada en la alimentación de @cont.bebenom?  Responde SÍ o NO a este mensaje |
| In English | Challenge: this week ask @cont.bebenom's dad, brother, cousin, grandpa or uncle to actively play together and have fun moments  Hello! Were you able to meet the challenge of getting @cont.bebenom's dad, brother, cousin, grandpa or uncle to actively play together? Respond YES or NO to this message  @cont.mamanom boys and girls from 2 to 5 years old should do physical activity or exercise until they reach 3 fun hours of play throughout the day | Challenge: this week prepare @cont.bebenom meals with vegetables, for example pumpkin, nopales, elotes or chayote. Try to include at least 1 vegetable a day  Hello @cont.mamanom! An excellent option for @cont.bebenom is to give him seasonal vegetables, because they are fresher and cheaper.  Hello @cont.mamanom, were you able to include seasonal vegetables in @cont.bebenom's diet? Respond YES or NO to this message |
| Supportive to PC | ¡Fantástico, @cont.mamanom! Con estas acciones, cada día la salud de toda la familia y de @cont.bebenom será la mejor | ¡Felicitaciones! Las verduras de temporada son la mejor opción para mantener a @cont.bebenom saludable |
| In English | Fantastic, @cont.mamanom! With these actions, every day the health of the whole family and @cont.bebenom will be the best | Congratulations! Seasonal vegetables are the best option to keep @cont.bebenom healthy |
