# Supplementary material for: Effectiveness and Implementation of a Text Messaging mHealth Intervention to Prevent Childhood Obesity in Mexico in the COVID-19 Context: Mixed Methods Study
Source: JMIR Mhealth Uhealth. 2024 Apr 9;12:e55509. doi: 10.2196/55509 (PMC11005909; doi:10.2196/55509)
Supplement: Multimedia Appendix 2 [file mhealth_v12i1e55509_app2.docx]

Multimedia Appendix 2. **Index of knowledge and practices on topics of interest, NUTRES**

|  | | | | | |
| --- | --- | --- | --- | --- | --- |
| Topic | Knowledge dimension | Number of Questions (number of correct answers) | Scoring per Correct Answer | Maximum score |  |
| Physical activity (PA) | PA concept | 1 (4) | 0.25 | 1 |  |
|  | PA benefits | 1 (9) | ~0.11 | 1 |  |
|  | PA recommendations | 1 (7) | ~0.14 | 1 |  |
|  | Screen time recommendations | 1(1) | 1 | 1 |  |
| Healthy Feeding (HF) | Characteristics of an unhealthy feeding | 1(10) | 0.10 | 1 |  |
|  | Characteristics of a healthy menu | 1(14) | ~0.071 | 1 |  |
|  | Characteristics of healthy drinks | 1(6) | ~0.166 | 1 |  |
|  | Characteristics of an unhealthy drinks | 1(15) | ~0.066 | 1 |  |
| TOPICS AND DIMENSIONS OF PRACTICES | | | | | |
| Topic | Practice dimension | Number of Questions | Scoring per question | Maximum score |  |
| Physical activity (PA) | Activities carried out in the last 7 days | 28 | 1 | 28 |  |
| Healthy Feeding (HF) | Food consumption in the previous day | 15 | 1 | 34 |  |
|  | Beverage consumption in the previous day | 19 | 1 |  |  |
