# Supplementary material for: Effectiveness and Implementation of a Text Messaging mHealth Intervention to Prevent Childhood Obesity in Mexico in the COVID-19 Context: Mixed Methods Study
Source: JMIR Mhealth Uhealth. 2024 Apr 9;12:e55509. doi: 10.2196/55509 (PMC11005909; doi:10.2196/55509)
Supplement: Multimedia Appendix 3 [file mhealth_v12i1e55509_app3.docx]

Multimedia Appendix 3. **Categories tree for coding testimonies of primary caregivers participants of NUTRES, 2021.**

| **Codes** | **Definitions** |
| --- | --- |
| **1. NUTRES Strategy**  1.1 Definition  1.2 PC Function  1.3 Recruitment and Discharge  1.4 Overview  1.5 Sentiments  1.6 Opinions on the environment  1.7 PS recommendations  1.8 Feedback on NUTRES messages  1.9 Message formats  1.10 Interest in receiving more messages  1.11 Recommendations | **All information related to the NUTRES strategy in general**  1.1 Any information that defines what NUTRES stands for.  1.2 Any information that defines your role within NUTRES.  1.3 Any information describing recruitment and dismissal.  1.4 Any information relating to the elements of NUTRES (infographics, videos, NUTRES equipment, most and least appreciated).  1.5 All information relating to any feeling/emotion towards NUTRES.  1.6 Any information that mentions the opinions and attitudes of the environment towards NUTRES.  1.7 Any information that mentions comments made by the SP in relation to issues and recommendations received with NUTRES.  1.8 General considerations about the NUTRES messages (clarity, relevance, culturally appropriate, easy to implement, etc.).  1.9 Any information that mentions message formats (favorite, least favorite, identified formats).  1.10 Any information related to interest in receiving more messages (duration, message topics, etc.).   - 1. 1.11 Any recommendations made by CPs to improve NUTRES**.** |
| **2. Mobile use**  2.1 Reading messages  2.2 Reading times  2.3 Reasons for NOT reading  2.4 SMS non-response  2.5 Problems encountered  2.6 Solution  2.7 WhatsApp feedback  2.8 Internet access | **All information related to the use of mobile phones by CPs.**  2.1 All information indicating whether all or some NUTRES messages have been read.  2.2 Any information indicating the times at which NUTRES messages were read.  2.3 Any reasons/justifications for not reading the NUTRES messages.  2.4 Any reasons/justifications for not replying to NUTRES messages.  2.5 Any problems related to mobile use reported by CPs.  2.6 Any information relating to the resolution of problems with the use of mobile phones (how, with the assistance of whom, etc.).  2.7 Any information related to the promotion of WhatsApp as a possible alternative.  2.8 Any information describing the type of internet access used by the CP. |
| **3. NUTRES Topics and Messages**  3.1 Retrieved topics and messages  3.2 Appreciation of themes and messages  3.3 Personal attitude  3.4 Benefits of messages  3.4.1 Knowledge  3.4.2 Perceived control  3.4.3 Perceived standard  3.4.4 Other  3.5 Implementation of recommendations  3.6 Non-implementation of recommendations | **All information related to the NUTRES themes and messages.**  3.1 Any information that mentions NUTRES themes and messages.  3.2 All information related to evaluations (positive and negative) of NUTRES themes and messages.3.3 All information related to CPs' personal attitudes towards NUTRES themes and messages.3.4 All information related to the perceived benefits of these NUTRES messages by the CPs.  3.5 All information related to the recommendations implemented (type, reason, etc.).  3.6 Any information related to the recommendations that were NOT implemented (type, reason, barriers, etc.). |
