# Supplementary material for: Effectiveness and Implementation of a Text Messaging mHealth Intervention to Prevent Childhood Obesity in Mexico in the COVID-19 Context: Mixed Methods Study
Source: JMIR Mhealth Uhealth. 2024 Apr 9;12:e55509. doi: 10.2196/55509 (PMC11005909; doi:10.2196/55509)
Supplement: Multimedia Appendix 4 [file mhealth_v12i1e55509_app4.docx]

Multimedia Appendix 4. Sociodemographic characteristics of primary caregivers at baseline and lost to follow-up. NUTRES, 2020-21.

|  | Lost to Follow up | | | | | Continue | | | | |
| --- | --- | --- | --- | --- | --- | --- | --- | --- | --- | --- |
|  | (n=64) | |  | |  | **(n=339)** | |  | |  |
|  |  | % or mean (±SD) |  |  |  |  | % or mean (±SD) |  |  | P Value * |
| **States of Mexico** |  |  |  |  |  |  |  |  |  |  |
| Morelos |  | 25.0 |  |  |  |  | 35.6 |  |  | 0,098 |
| Yucatán |  | 75.0 |  |  |  |  | 64.3 |  |  |  |
| Area |  |  |  |  |  |  |  |  |  |  |
| Urban |  | 67.1 |  |  |  |  | 38.6 |  |  | 0,377 |
| Rural |  | 32.8 |  |  |  |  | 61.3 |  |  |  |
| **Age of primary caregiver** |  | 26.7 (±7.38) |  |  |  |  | 27.8(±6.82) |  |  | 0,224 |
| Relationship to child |  |  |  |  |  |  |  |  |  |  |
| Mother |  | 100.0 |  |  |  |  | 98.8 |  |  | 0,948 |
| Grandmother |  | 0 |  |  |  |  | 0.88 |  |  |  |
| Father/grandfather |  | 0 |  |  |  |  | 0.29 |  |  |  |
| **Sex of child** |  |  |  |  |  |  |  |  |  |  |
| Girl |  | 46.8 |  |  |  |  | 44.8 |  |  | 0,504 |
| -Boy |  | 53.1 |  |  |  |  | 53.1 |  |  |  |
| Age of child (months) |  | 18.3 (±17.5) |  |  |  |  | 19.1(±16.6) |  |  | 0,753 |
| <24 |  | 65.6 |  |  |  |  | 66.0 |  |  | 0,944 |
| 24-59 |  | 34.3 |  |  |  |  | 33.9 |  |  |  |
| **Marital status (married/free union)** |  | 77.7 |  |  |  |  | 88.7 |  |  | 0,133 |
| Schooling (basic or less) |  | 0 |  |  |  |  | 12.0 |  |  | 0,613 |
| **Employment with payment (last week) (yes)** |  | 11.1 |  |  |  |  | 28.0 |  |  | 0,263 |
| **Socioeconomic Status (SES) ^a^** |  |  |  |  |  |  |  |  |  |  |
| Tertile 1 |  | 25.0 |  |  |  |  | 33.6 |  |  | 0,591 |
| Tertile 2 |  | 50.0 |  |  |  |  | 32.7 |  |  |  |
| Tertile 3 |  | 25.0 |  |  |  |  | 33.6 |  |  |  |
| **Beneficiary/affiliation social program (yes)** |  | 11.1 |  |  |  |  | 26.2 |  |  | 0,581 |
| SD: standard deviation; **^a^** Socioeconomic Status (tercile 1 represents the lowest welfare conditions) | | | | | | | | | | |
