# Supplementary material for: Effectiveness and Implementation of a Text Messaging mHealth Intervention to Prevent Childhood Obesity in Mexico in the COVID-19 Context: Mixed Methods Study
Source: JMIR Mhealth Uhealth. 2024 Apr 9;12:e55509. doi: 10.2196/55509 (PMC11005909; doi:10.2196/55509)
Supplement: Multimedia Appendix 5 [file mhealth_v12i1e55509_app5.docx]

Multimedia Appendix 5. **Main characteristics of primary caregivers interviewed. NUTRES, 2020-21.**

| **Interview reference** | **State** | **Type of locality** | **Level of interactivity** | **Age of the PC** | **Marital status** | **Level of schooling** | **Occupation** | **Relationship to child in the study** | **Age of child** | **Range of children** |
| --- | --- | --- | --- | --- | --- | --- | --- | --- | --- | --- |
| #1 | Yucatán | Urban | Low | 34 | Married | University | Housewife | Mother | 9-11 meses | 3 |
| #2 | Yucatán | Urban | Low | 25 | Single | University | Worker/Employee | Mother | 6-8 meses | 1 |
| #3 | Yucatán | Urban | High | 22 | Single | University | Housewife | Mother | 9-11 meses | 1 |
| #4 | Yucatán | Rural | Low | 29 | Married | Middle high school | Housewife | Mother | 0-5 meses | 2 |
| #5 | Yucatán | Rural | High | 27 | Married | High school | Housewife | Mother | 0-5 meses | 1 |
| #6 | Yucatán | Rural | Low | 23 | Married | Middle high school | Housewife | Mother | 0-5 meses | 2 |
| #7 | Yucatán | Urban | Low | 39 | Married | Middle high school | Worker/Employee | Mother | 2-5 años | 3 |
| #8 | Yucatán | Urban | Low | 32 | Married | Basic | Worker/Employee | Mother | 2-5 años | 4 |
| #9 | Yucatán | Urban | High | 30 | Separated | Middle high school | Worker/Employee | Mother | 2-5 años | 1 |
| #10 | Yucatán | Urban | Low | 30 | Married | High school | Housewife | Mother | 2-5 años | 3 |
| #11 | Yucatán | Urban | Low | 37 | Married | Middle high school | Housewife | Mother | 2-5 años | 1 |
| #12 | Yucatán | Rural | High | 32 | Married | University | Housewife | Mother | 2-5 años | 2 |
| #13 | Morelos | Urban | Low | 38 | Married | Middle high school | Housewife | Mother | 6-8 meses | 4 |
| #14 | Morelos | Urban | Low | 16 | Single | Middle high school | Housewife | Mother | 0-5 meses | 1 |
| #15 | Morelos | Urban | High | 32 | Cohabiting relationship | High school | Housewife | Mother | 6-8 meses | 2 |
| #16 | Morelos | Rural | Low | 34 | Cohabiting relationship | High school | Housewife | Mother | 0-5 meses | Doesn´t Know |
| #17 | Morelos | Rural | High | 19 | Cohabiting relationship | Middle high school | Worker/Employee | Mother | 0-5 meses | N/A |
| #18 | Morelos | Rural | Low | 24 | Cohabiting relationship | Technical | Worker/Employee | Mother | 1-2 años | 15 |
| #19 | Morelos | Urban | High | 28 | Cohabiting relationship | High school | Housewife | Mother | 2-5 años | 1 |
| #20 | Morelos | Urban | Low | 28 | Cohabiting relationship | High school | Housewife | Mother | 2-5 años | 1 |
| #21 | Morelos | Urban | Low | 34 | Married | Middle high school | Worker/Employee | Mother | 2-5 años | 1 |
| #22 | Morelos | Rural | Low | 29 | Married | High school | Housewife | Mother | 2-5 años | 1 |
| #23 | Morelos | Rural | High | 41 | Married | University | Worker/Employee | Mother | 2-5 años | 3 |
| #24 | Morelos | Rural | Low | 39 | Married | High school | Housewife | Mother | 2-5 años | 4 |
