# Supplementary material for: Effectiveness and Implementation of a Text Messaging mHealth Intervention to Prevent Childhood Obesity in Mexico in the COVID-19 Context: Mixed Methods Study
Source: JMIR Mhealth Uhealth. 2024 Apr 9;12:e55509. doi: 10.2196/55509 (PMC11005909; doi:10.2196/55509)
Supplement: Multimedia Appendix 6 [file mhealth_v12i1e55509_app6.docx]

| **English quotes** | **Spanish quotes** |
| --- | --- |
| **Acceptance** | |
| *Well, I don't know, I say how.... one year, I don't know... because I still don't feel as prepared for... as for... well... yes, well yes, I do feel prepared for my baby, but I still don't know how many things, so it does help me* [#14]*.*  *They make me feel calm [the messages]. (S) how do I explain it? They make me feel calm because they are helping me” (...) and it helped me a lot with the advice is reaching me (...)* [#15].  *Mmm, well, it's a program that helps us, helps us complement what we already do at home for the children* [#24]. | *Pues no sé, yo digo como....un año, no sé...porque todavía no me siento tan preparada yo de...como de...pues...sí, ósea sí, si me siento preparada de mi bebé, pero como que todavía no sé cómo que muchas cosas, entonces pues sí me ayuda* [#14].  *Me hacen sentir como tranquila [los mensajes]. (S) ¿cómo le explico?, me hacen sentir tranquilidad porque pues sí me están ayudando” (...) y a mí me ayudó mucho con los consejos que me están llegando”) (...)* [#15].  *Mmm pues, es un programa que nos ayuda para, nos ayuda a complementar lo que nosotros ya hacemos en la casa para los niños* [#24]. |
| **Pertinency** | |
| *Since I am a first-timer, I felt that it helped me in the sense that, like right now, because of the Covid, there is no need for you to take, for example, to your health clinic, so there they told you how the processes that touched them...in what month could you give certain foods to your baby* [#5].  *Yes, because they were at the age, they were perfectly fine at my son's age. Hey? forever! [#13].* | *Como soy primeriza, sentí que me ayudó en el sentido de que, en como ahorita, por el Covid no hay de que tú lleves, por ejemplo, a tu clínica de salud, entonces ahí te decían como los procesos que les tocaban...en qué mes podrías darle ciertos alimentos a tu bebé* [#5]*.*  *Sí, porque iban a la edad, iban perfectamente bien a la edad de mi hijo. ¿eh? ¡siempre!* [#13]*.* |
| **Coverage** | |
| *Good. Well, in fact, we had to answer “Yes, No”, or if the goal was achieved or not, I tell him. There were times that the same and maybe I did not realize some messages because there was no coverage, they did not enter. Sometimes several days passed, and until I went out to some place that did exist, I was aware of the messages* [#23]*.*  *And I told him “it's that they don't reach me”, that only when I go to a higher part or I left here, they began to reach me. And I tell him, when I'm away, well, yes, I answer them, yes or...like no or something, well, the things they asked me* [#21]*.*  *Here in the town the current fails a lot, and the signal goes away...it's been like [P] four months now that the current goes out constantly here in the town and it's in parts* [#6].  *Well, almost all of them because it was at the beginning when I began to receive the messages that they were going to charge me. And then, well, this...well, my recharge ran out very quickly, so to speak, and I preferred to just read them and put them into practice...Sometimes I would send them and say no, that I had to charge I don't know what...things like of money or something like that, and others if they let them answer* [#14].  *What happens, that as there was a problem, that the telephones were lost. There came a time when this number that I have, my husband had it, and since we removed the chip, it disappeared* [#5]. | *Bien. Pues, de hecho, teníamos que responder “Sí, No”, o si se lograba la meta o no se lograba, le digo. Había ocasiones que igual y a lo mejor no me llegué a dar cuenta de algunos mensajes porque no había cobertura, no entraban. A veces pasaban varios días, y hasta que llegaba a salir a algún lugar que sí había, me daba cuenta de los mensajes* [#23]*.*  *Y le dije “es que no me llegan”, que solamente que cuando salgo a una parte más alta o salía fuera de acá, me empezaban a llegar. Y le digo, cuando estoy fuera, pues sí ya les contesto, que sí o que...como que no o algo, pues las cosas que me preguntaban* [#21]*.*  *Aquí en el pueblo falla mucho la corriente, y se va la señal...ya tiene como [P] cuatro meses que constante se va la corriente aquí en el pueblo y es por partes* [#6].  *Pues casi todos porque fue al principio cuando me empezó a llegar lo mensajes de que me iban a cobrar. Y entonces, pues, este...pues a mí se me acababa muy rápido mi recarga, por decir así y prefería mejor namás leerlos y ponerlos en práctica...A veces los mandaba y que decía no, que tenía que cobrar no sé qué...cosas como de dinero o algo así, y otros si los dejaban contestar* [#14]*.*  *Lo que pasa, que como hubo un problema, que se echaron a perder los teléfonos. Llegó un momento en que este número que tengo, mi esposo lo tenía, y como quitamos el chip, desapareció* [#5]*.* |
